# Does the Performance of a Six-Minute Walking Test Predict Cardiopulmonary Complications After Uniportal Video-Assisted Thoracic Surgery Anatomic Lung Resection?

**DOI:** 10.3390/cancers17010032

**Published:** 2024-12-26

**Authors:** Michele Salati, Marco Andolfi, Alberto Roncon, Gian Marco Guiducci, Francesco Xiumè, Michela Tiberi, Anna Chiara Nanto, Sara Cingolani, Eleonora Ricci, Majed Refai

**Affiliations:** 1 Unit of Thoracic Surgery, AOU of Marche, 60126 Ancona, Italyannachiara.nanto@ospedaliriuniti.marche.it (A.C.N.);; 2 Unit of Rehabilitation Medicine, AOU of Marche, 60126 Ancona, Italy

**Keywords:** 6MWT, uniportal VATS, minimally invasive surgery, lung resection, risk assessment, complications, pre-habilitation

## Abstract

Objectives: The purpose of the present study was to verify if performance in the 6-min walking test (6MWT) during the preoperative evaluation phase is associated with the development of cardiopulmonary postoperative complications in patients who underwent uniportal VATS (U-VATS) for lung cancer. Methods: This retrospective, monocentric study included patients submitted to U-VATS anatomical lung resections (March 2022–December 2023). The patients were enrolled in a preoperative rehabilitation program carried out 15 days before surgery. The 6MWT was performed at counseling (T0) and after pre-habilitation (T1). Univariate analysis followed by logistic regression verified the association of baseline patients’ characteristics and performance in the 6MWT (meters walked during T0 and T1 and the difference between T1 and T0—T1-T0 variation) with postoperative cardiopulmonary complications (CPCs). Youde’s index was used to establish the optimal cut-offs for ergometric parameters significantly correlated with CPCs. Results: We enrolled 212 patients scheduled to undergo U-VATS lung resection (lobectomies: 177; bilobectomies: 2; segmentectomies: 33). Twenty-three (10.8%) patients developed CPCs. None of the baseline patients’ characteristics were associated with CPCs. Complicated patients showed more significant differences compared to non-complicated ones for meters walked during the 6MWT T1 (6MWT-T1-complicated: 450 vs. 6MWT-T1-non-complicated: 517; *p*: 0.01) and for variation-T1-T0 (variation-T1-T0-complicated: 4 m vs. variation-T1-T0-non-complicated: 20 m; *p*: 0.02). The best cut-offs for discriminating between patients with CPCs and those with uneventful courses were 458 m for 6MWT-T1 and 31 m for variation-T1-T0. After multivariate analysis, 6MWT-T1 < 458 m and variation-T1-T0 < 31 m were the unique parameters independently correlated with CPCs (*p*: 0.03 and *p*: 0.05, respectively). Conclusions: The 6MWT results (in particular, 6MWT-T1 < 458 m and variation-T1-T0 < 31 m) in the context of a pre-habilitation program are associated with the development of CPCs after U-VATS lung resection.

## Simple Summary

At present, the functional assessment algorithms used to evaluate fitness for surgical treatment for lung cancer are mostly focused on candidates for open thoracic surgery. Updated evidence aimed at defining complication predictors in lung cancer patients undergoing curative video-assisted thoracic surgery (VATS) could refine the risk assessment strategies in current clinical practice. This retrospective study verified if performance in the 6-min walking test (6MWT) was associated with cardiopulmonary postoperative complications in patients who underwent uniportal VATS lung resection for cancer. Two ergometric parameters obtained using the 6MWT (6MWT-T1 < 458 m and T1–T0 variation < 31 m) proved to be unique independent factors associated with a complicated postoperative course. Patients with a poor 6MWT performance experienced a three- to four-fold higher rate of postoperative cardiopulmonary complications. The 6MWT should be considered a valuable tool for measuring risk after uniportal VATS anatomic lung resection, and its adoption is recommended in the preoperative evaluation of patients’ fitness for surgery.

## 1. Introduction

Anatomic lung resection is recommended in cases of early-stage neoplasm for patients with lung cancer [1,2]. However, the chance of offering this therapeutic option is subject to individuals’ fitness for surgery. As a consequence, the assessment of complications and mortality risk in cases of operation is pivotal in the preoperative evaluation of lung resection candidates.

To this end, several algorithms have been published by international societies aimed at defining the optimal preoperative functional assessment pathway for lung resection candidates as well as at identifying those factors and conditions considered contraindications for surgical treatment [3,4]. The functional parameters commonly used to define the risk of complications in cases of lung surgery are obtained by spirometry assessment (in particular, the forced expiratory volume in one second—FEV1; the diffusing capacity for carbon monoxide—DLCO; and their correlated postoperative predicted values) by low-tech exercise tests (such as meters climbed in the stair-climbing test or meters walked in the 6-min walking test—6MWT) and by performance measured with a cycle ergo spirometer, which directly measures the maximal oxygen consumption—VO_2_max—of patients. In addition, other risk factors for adverse events to take into account before lung resection have been identified through studies performed on large international multi-institutional databases and aggregated into specific risk scores, including patient age, ASA score, thoracotomy approach, pulmonary resections extended to other structures (i.e., chest wall, atrium or superior vena cava, diaphragm, vertebrae, etc.), and the presence of other comorbidities (such as chronic artery disease, cerebrovascular disease, and chronic kidney disease) [5,6,7].

The research that identified these risk factors and the above-mentioned guidelines that provided scientific evidence to support clinicians in the process of risk assessment was published more than ten years ago. Moreover, these studies were mostly based on cohorts of patients treated using the open surgical technique through the thoracotomy approach.

This could represent a limiting background to the current clinical practice, considering that nowadays, many centers are offering minimally invasive anatomic lung resection as the standard of care to patients affected by lung cancer, as is largely recommended [1,8,9,10]. Moreover, this minimally invasive approach is often integrated into pathways of care designed for minimizing the impact of surgery and achieving a fast and uneventful recovery after lung resection [11,12,13].

The aim of the present study was to define the role of performance in the 6MWT as a risk factor for the development of cardiopulmonary complications in a cohort of patients submitted to anatomic resection by video-assisted thoracic surgery (VATS) for non-small cell lung cancer (NSCLC) in the context of an enhanced pathway of care (EPC) program.

## 2. Materials and Methods

In the present study, we analyzed a consecutive series of 212 patients affected by NSCLC who were undergoing uniportal VATS (U-VATS) anatomic lung resection at our institution from March 2022 to December 2023.

The indication to the surgical treatment was defined during dedicated meetings by a multidisciplinary working group, including thoracic surgeons, oncologists, pulmonologists, radiologists, and pathologist representatives.

The baseline characteristics and medical history of each patient were assessed during the preoperative visit 15 to 20 days before the operation. In the same visit, the patients underwent cardiac evaluation by the cardiologist, a pulmonary function test with the measurement of carbon monoxide lung diffusion capacity (DLCO), and a preoperative anesthesiologist assessment. Moreover, they were evaluated by a physiatrist and physiotherapist who explained the exercises included within the pre-habilitation program and submitted each patient to the 6MWT (as described below at Section 2.1).

The functional evaluation to define the patients’ fitness according to the proposed surgical treatment was performed following international published protocols [3,4]. We considered contraindications to anatomic lung resection a non-stable hearth disease, a predicted postoperative forced expiratory volume in 1 s (ppoFEV1), and/or a predicted postoperative DLCO (ppoDLCO) lower than 30% with a VO2 peak of less than 10 mL/kg/min. Both ppoFEV1 and ppoDLCO were calculated by considering the number of functioning segments to remove during operation and estimated by means of bronchoscopy and computed tomography (CT) scan or using the ventilation/perfusion lung scan, if necessary.

The patients’ operative and postoperative management was standardized using the institutional EPC protocols as elsewhere described [14,15]. The main strategies adopted are summarized in Table 1.

The present study was submitted for examination and approval to the responsible review board the Ethical Committee of Marche Region.

This study involving human participants was performed in accordance with the tenets of the Declaration of Helsinki and according to national regulations. Written informed consent was obtained from all subjects involved in the study.

### 2.1. Study Design: 6MWT and Outcomes

Patients were considered eligible for the present study if ≥18 years of age, an American Society of Anesthesiologists score ≤ 3, and scheduled for elective anatomic lung resection for NSCLC. Patients were excluded if at least one of these conditions were present: American Society of Anesthesiologists score > 3, conversion to open surgery, planned postoperative intensive-care monitoring, lack of caregiver, inability to collaborate (language barriers and psychiatric pathology), or physical impairment that affects walking.

**Figure 1:**
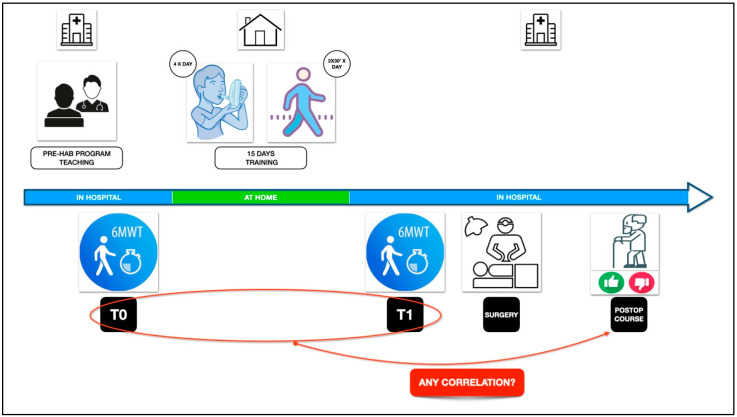
Visual study design outlining the main logical points of the present research that tested a potential correlation between the ergometric performance of the patients and their postoperative course.

The logic structure of the present study is visualized in Figure 1. The pre-habilitation program was presented to all patients enrolled in the present study during the preoperative visit. They received instructions from the physiotherapist about the use of the incentive spirometry and the exercises comprising the “controlled breathing cycle” (a progressive chest wall expansion followed by forced expiration and the cough stimulation). Then, the patients were commissioned to perform the pre-habilitation training protocol at home, including 4 sessions per day of incentive spirometry and breathing cycles combined with 2 sessions per day of 30 min free-walking. The program was maintained for 15 days before surgery.

In order to measure the potential cardiopulmonary improvement consequent to the training, we adopted a low-tech exercise test already validated for the physical assessment before thoracic surgery as the 6MWT [16,17]. The 6MWT was delivered according to the American Thoracic Society guidelines [18] and after the cardiologic evaluation, which excluded potential contraindication to exercise testing (no patients were excluded from the 6MWT in the present series). All the patients performed the 6MWT at baseline during the preoperative visit (T0) and 1 day before surgery (T1). No complications related to the 6MWT were registered. The meters walked for T0 (6MWT-T0) and the meters walked for T1 (6MWT-T1) after the completion of the pre-habilitation as well as the difference between the meters walked for T1 and T0 (variation T1-T0), which measures the gain obtained with the pre-habilitation, have been tested for an association with the postoperative cardiopulmonary complications (CPCs) rate.

Moreover, we tested the association of several preoperative baseline patients characteristics (age, sex, body mass index, FEV1, ppoFEV1, DLCO, ppoDLCO, Tiffenau index, chronic obstructive pulmonary disease index, pack-years, ASA score, comorbidities, and type of resection) with the occurrence of postoperative cardiopulmonary complications (CPCs) after uniportal VATS anatomic lung resection.

The following CPCs with standardized definitions reported in the literature [19] have been considered for the present study: pneumonia, atelectasis requiring bronchoscopy, respiratory failure, reintubation, acute respiratory distress syndrome, pulmonary embolism, pulmonary edema, acute myocardial ischemia, arrhythmia, acute cardiac failure, stroke or transient ischemic attack, and acute kidney disease. We evaluated the complications that arose within the first 30 days after the operation and/or during the entire hospital stay.

The uniportal VATS procedure was performed through a single 3–4 cm incision at the 5th intercostal space on the anterior axillary line, as elsewhere reported [14,20]. After the anatomic lung resection, the systematic nodal dissection was performed in all patients. During the study period, the uniportal VATS represented the standard procedure systematically adopted for treating all the patients affected by lung neoplasms. As a consequence, 97% of the patients were treated by uniportal VATS, with 3% being the conversion rate to open the procedure (the patients operated on by open surgery were excluded from the present study).

### 2.2. Statistical Analysis

Data for the present analysis were retrieved from our institutional prospectively maintained database. Before analysis data underwent data quality assessment and data cleaning procedure, variables with a completeness rate < 90% as well as patients with lacking data about one of the outcomes were excluded from the analysis. Specific imputation techniques were used for cleaning variables with up to 10% incompleteness.

Numeric variables were tested for normality by the Shapiro–Wilk test. The student *t* test was used to test numeric variables with normal distribution, and the Mann–Whitney test for those without normal distribution. The chi-square test was used to test categoric variables (no variable with less than 10 observations in 1 cell). The univariate analysis tested the possible association of several preoperative baseline characteristics of the patients as well as their performance during the 6MWT with postoperative CPCs.

Youden’s index [21] was used to establish the best cut-off values for those ergometric parameters significantly correlated to CPCs (in particular, 6MWT T1 and variation T1-T0) in order to more simply apply these thresholds for the definition of the surgical risk in the clinical practice.

Those parameters with a *p* ≤ 0.1 at the univariate analysis were then included in a stepwise logistic regression to identify independent predictors of CPCs.

Statistical analysis was performed on the statistical software Stata 14.0 (StataCorp, LP, College Station, TX, USA).

## 3. Results

During the study period, we enrolled 212 patients undergoing U-VATS anatomic lung resection and trained following the pre-habilitation program. We excluded four patients from the study that were considered unsuitable for the training and the 6MWT due to physical impairment. Looking at the 212 analyzed patients, the mean age was 67.9 years, and the majority was represented by men (male patients 53.8%). In this cohort, 177 patients (83.5%) underwent lobectomy, and 2 (1%) bilobectomy and 33 (15.5%) underwent segmentectomy. The baseline characteristics as well as the performance results from the 6MWT of the entire population are summarized in Table 2. The meters walked during the 6MWT during the first evaluation (mean 6MWT-T0: 491.6 m) improved on average by 23.4 m after the training.

None of the patients’ baseline characteristics tested during the univariate analysis were associated with cardiopulmonary complications (Table 3).

**Figure 2:**
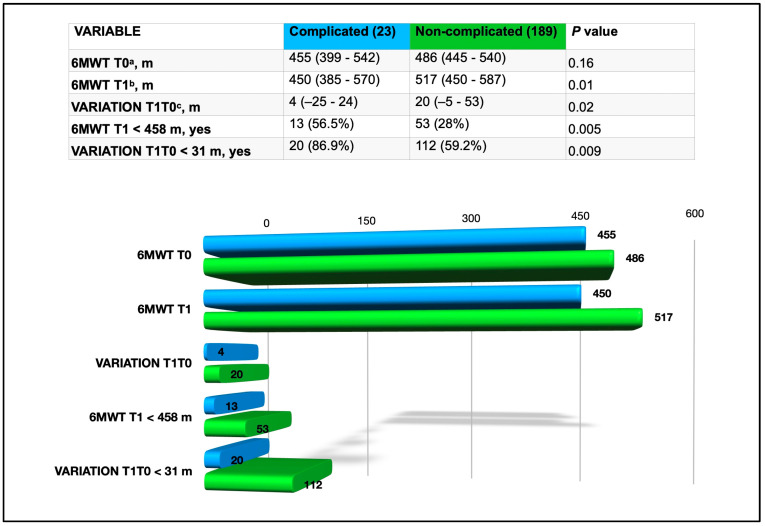
Results comparison of the ergometric performance in the 6MWT between complicated and non-complicated patients in terms of meters walked during the preoperative visit (6MWT T0) and the day before surgery (6MWT T1), difference between the meters walked during the 6MWT at T1 and T0 (VARIATION T1T0) and considering the specific cut offs for the 6MWT T1 and for the VARIATION T1T0. ^a^ 6MWT T0: six-min walking test at baseline during the preoperative visit, values are median (interquartile range). ^b^ 6MWT T1: six-min walking test one day before surgery, values are median (interquartile range). ^c^ VARIATION T1T0: difference between the meters walked during the 6MWT for T1 and T0, values are median (interquartile range).

On the other hand, considering the results of the 6MWT (Figure 2), the meters walked during the 6MWT-T1 and the T1-T0 variation were significantly correlated to the cardiopulmonary complications at the univariate analysis.

Table 4 shows the cardiopulmonary complications rate for each type of operation and their granular distribution.

The optimal cut-offs for discriminating patients with cardiopulmonary complications from those with uneventful course were 458 m for the 6MWT-T1 (AUC 0.64; sensitivity 0.57; specificity 0.72) and 31 m for the T1-T0 variation (AUC 0.64; sensitivity 0.87; specificity 0.51. A poor performance during the 6MWT for T1 (6MWT-T1 < 458 m) and a limited improvement of the walked distance after the training (T1-T0 variation < 31 m) were also associated with postoperative cardiopulmonary complications, as reported in Figure 2.

After the stepwise logistic regression, including those factors with a *p*-value ≤ 0.1 according to univariate analysis (age, DLCO, ppoDLCO, coronary artery disease, 6MWT-T1, T1-T0 variation, 6MWT-T1 < 458 m, T1-T0 variation < 31 m), the independent parameters associated with the cardiopulmonary complications were 6MWT-T1 < 458 m and T1-T0 variation < 31 m (Table 5).

**Figure 3:**
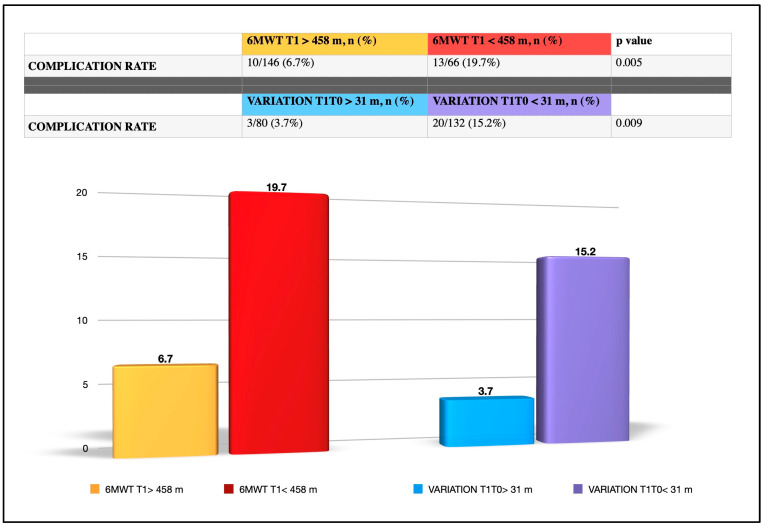
Complications rate and performance during 6MWT using specific cut-offs for the six-min walking test performed one day before surgery (6MWT T1) and for the difference between the meters walked during the 6MWT for T1 and T0 (VARIATION T1T0).

Figure 3 shows the different rates of cardiopulmonary complications in patients with 6MWT-T1 < 458 m and T1-T0 variation < 31 m in comparison to their counterparts with a better ergonometric performance.

## 4. Discussion

The present study shows that the performance obtained during the 6MWT test is associated with the risk of experiencing cardiopulmonary complications during the postoperative course in patients submitted to video-assisted anatomic lung resection for lung cancer.

In particular, we found that walking a distance of less than 458 m during the 6MWT after the completion of the training program is predictive of postoperative cardiopulmonary complications. This finding seems in line with some previous studies published in the literature aimed at verifying the role of the 6MWT in predicting complications after lung resection [22,23,24]. Recently, Marjanski et al. reported the results of a study on 253 patients submitted to lobectomy who performed the 6MWT the day before surgery [25]. They found that the patients who reached less than 500 m during the 6MWT (a threshold a bit higher than the one in our experience) as well as those with a lower FEV1 had an increased risk of postoperative cardiopulmonary complications and prolonged hospital stays. But, in their study, only 22% of the patients were operated on by VATS. We focused our research only on patients submitted to VATS resections because in our current practice, more than 95% of lung cancer patients are treated by minimally invasive anatomic resection, in particular using the uniportal VATS technique. For this group of surgical patients, we identified a specific limit of the 6MWT set at 458 m that could represent a parameter to be tested by future studies as a reliable predictor of cardiopulmonary complications after VATS lung resection. In our experience, those patients that walked more than 458 m during the T1 6MWT experienced a cardiopulmonary complication rate of 6.8% vs. 17.8% of the counterparts that walked a lower distance.

Moreover, thanks to the pre-habilitation program, we had the chance to compare the patients’ performance during the 6MWT before and after the training. We found that an improvement lower than 31 m from the 6MWT-T0 to 6MWT-T1 is another parameter strongly associated with the development of postoperative cardiopulmonary complications.

There are indeed several studies that have demonstrated the effectiveness of a presurgical training in increasing the ergometric performance measured through the 6MWT of candidates to lung resection for lung neoplasms [26,27]. Nevertheless, it is not clear if this enhanced aerobic condition translates into better clinical outcome and, in particular, into a lower rate of postoperative complications [28,29,30]. Moreover, these studies are performed on small cohorts composed mostly of patients operated on by the open approach.

In our research, we found that obtaining an improvement of the walking meters after training lower than 31 m is an independent risk factor for experiencing postoperative cardiopulmonary complications. So much so that those patients with a T1-T0 6MWT < 31 m had a rate of cardiopulmonary complications more than three times higher (T1-T0 6MWT > 31 m complications rate: 3.75% vs. T1-T0 6MWT < 31 m complication rate: 14.39%, *p*: 0.01) than the counterparts with a grater improvement.

Finally, even though the preoperative assessment for defining the patients’ operability is based on the algorithms derived from the current international guidelines [3,4], we failed to find a correlation between the traditional risk factors and the postoperative outcomes in our cohort. In our study, the distance walked for the T1 6MWT and the difference between the meters obtained for the T1 6MWT vs. T0 6MWT were the only predictors of cardiopulmonary complications for patients submitted to U-VATS anatomic lung resection after the logistic regression, questioning the role of indicators such as FEV1, DLCO, age, BMI, ASA score, and cardiac comorbidities, which are usually used for assessing the surgical risk for lung resection performed through the open approach.

### Limits

The present study has some limitations. First of all, the 6MWT is a submaximal exercise test and its correlation to tools for direct maximal cardiopulmonary functional assessment as the cycle ergo spirometer is not univocally defined. Although, the use of the results of the 6MWT for assessing the risk of surgical resection should be further validated by formal CPET, especially for those patients with a low 6MWT performance (walking distance lower than 458 m).

Moreover, the 6MWT cannot be administered to patients with cardiovascular contraindications for exercise tests, as well as to patients with physical impairments concerning the ability to walk. In our study, four patients did not perform the 6MWT.

Another limit of this analysis is represented by the need to verify result generalizability in different clinical settings. In fact, we performed the 6MWT in the context of a pre-habilitation program that needs the cooperation of physiatrists, which both explains the training program and measures its effectiveness through the 6MWT for T0 and T1. Furthermore, the preoperative, intraoperative, and postoperative phases of the clinical course in our unit are standardized and organized following EPC protocols, which could have influence in the obtained outcomes.

## 5. Conclusions

We have demonstrated the potential role of the 6MWT in the pre-surgical workup aimed at evaluating the risk of complicated postoperative courses in patients submitted to anatomic lung resection by VATS. We would recommend the systematic adoption of this low-tech exercise test before minimally invasive lung resection in order to complement the risk assessment performance of other risk factors previously identified for open surgery candidates. The results of the 6MWT could support patients and caregivers for a more informed decision with regard to surgical therapy, as well as the physicians in planning the optimal postoperative pathway of care, especially for subjects at high risk of complications.

## Figures and Tables

**Table 1:** Main strategies adopted in the institutional enhanced pathway of care.

Intraoperative Phase
Standardized antibiotic prophylaxis
Intraoperative hypothermia prevention
Minimization of vascular accesses and catheters
Surgical technique: uniportal VATS
Intraoperative air leak assessment at the end of the surgical procedure and adoption of control measures (sutures reinforcement and use of sealants)
Standardization of anesthetic techniques (preservation of stable body temperature, opioid-free multimodal analgesic therapy with specific loco-regional anesthetic techniques such as paravertebral thoracic (T5) erector spinae plane block, and fluid therapy control)
Postoperative phase
Early mobilization (4 to 6 h after surgery)
Standardization of thromboembolic prophylaxis
Standardization of the analgesic technique (titrated to keep the pain visual score below 4 on a scale of 0 to 10) switching from fractionated intravenous to oral painkiller administration as soon as possible
Oral intake of fluid and solid foods during the day of operation (6 h after surgery)
Daily sessions of respiratory physiotherapy monitored by dedicated physiotherapist (twice a day)
Quick removal of vascular accesses and catheters
Chest tube removal in the second postoperative day (if no air leak was registered during the previous 12 h by a digital chest drainage system and pleural effusion was less than 400 mL/24 h)

**Table 2:** Baseline characteristics and 6MWT results of patients.

Variable	All Patients (212)
AGE, y	69 (63–74)
SEX, Male	114 (53.8)
BMI, kg/m^2^	25.8 (23.6–29)
FEV1%	96 (84.5–107)
ppoFEV1%	79 (67.8–89.5)
DLCO	75 (65–86)
ppoDLCO	61.2 (52.3–70.3)
TIFF	0.73 (0.67–0.78)
COPD, yes	75 (35.4)
PACK YEARS	20 (0–40)
ASA	1 (1–2)
CAD, yes	23 (10.8)
CVD, yes	12 (5.7)
CKD, yes	16 (7.5)
CLD, yes	12 (5.7)
DIA, yes	24 (16.0)
Type of resection	
Lobectomy	177 (83.5)
Bilobectomy	2 (1.0)
Segmentectomy	33 (15.5)
6MWT T0, m	485 (441–540)
6MWT T1, m	551 (450–587)
VARIATION T1T0, m	20 (−7.5–50)
6MWT T1 < 458 m, yes	66 (31.1)
VARIATION T1T0 < 31 m, yes	132 (66.4)

Values are median (interquartile range), No. (%), or as otherwise indicated. BMI: body mass index, FEV1: forced expiratory volume in 1 s, ppoFEV1: predicted postoperative FEV1, DLCO: carbon monoxide lung diffusion capacity, ppoDLCO: predicted postoperative DLCO, TIFF: Tiffenau index, COPD: chronic obstructive pulmonary disease, ASA: American Society of Anesthesiologists, CAD: coronary artery disease, CVD: cerebrovascular disease, CKD: chronic kidney disease, CLD: chronic liver disease, DIA: diabetes. 6MWT T0: six-min walking test at baseline during the preoperative visit, 6MWT T1: six-min walking test one day before surgery, VARIATION T1T0: difference between the meters walked during the 6MWT for T1 and T0.

**Table 3:** Univariate analysis comparing complicated and non-complicated patients.

Variable	Complicated (23)	Non-Complicated (189)	*p* Value
AGE, y	74 (66–76)	68 (62–74)	0.09
SEX, Male	14 (60.9)	100 (52.9)	0.47
BMI	25.3 (23.5–29.8)	25.9 (23.6–29)	0.64
FEV1%,	96 (86–110)	96 (84–107)	0.99
ppoFEV1%	76.8 (68.2–88.4)	79.2 (67.6–89.7)	0.51
DLCO	70 (64–78)	76 (65–87)	0.08
ppoDLCO	57.3 (50.1–67.1)	61.5 (53.4–70.7)	0.07
TIFF	0.73 (0.64–0.8)	0.73 (0.68–0.78)	0.81
COPD, yes	8 (34.5)	67 (35.4)	0.89
PACK YEARS	30 (0–45)	20 (0–40)	0.83
ASA	1 (1–2)	1 (1–2)	0.52
CAD, yes	1 (4.3)	22 (11.6)	0.08
CVD, yes	3 (13)	9 (4.8)	0.11
CKD, yes	3 (13.1)	13 (6.9)	0.29
CLD, yes	1 (4.3)	11 (5.8)	0.77
DIA, yes	5 (21.7)	29 (15.5)	0.43
Type resection			0.54
Lobectomy	21 (91.3)	156 (82.5)	
Bilobectomy	0 (0)	2 (1.1)	
Segmentectomy	2 (8.7)	31 (16.4)	

Values are median (interquartile range), No. (%), or as otherwise indicated. BMI: body mass index, FEV1: forced expiratory volume in 1 s, ppoFEV1: predicted postoperative FEV1, DLCO: carbon monoxide lung diffusion capacity, ppoDLCO: predicted postoperative DLCO, TIFF: Tiffenau index, COPD: chronic obstructive pulmonary disease, ASA: American Society of Anesthesiologists, CAD: coronary artery disease, CVD: cerebrovascular disease, CKD: chronic kidney disease, CLD: chronic liver disease, DIA: diabetes.

**Table 4:** Distribution of the cardiopulmonary complications sorted by type of operation.

Type of Operation	Complicated Patients (n)	Non-Complicated Patients (n)	Complication Rate	Type of Complications
Lobectomy	21	156	11.9%	9 postoperative arrhythmia 7 pneumonia 2 atelectasis 1 acute myocardial ischemia 1 acute kidney injury 1 pulmonary embolism 1transient ischemic attack
Segmentectomy	2	31	6%	2 postoperative arrhythmia
Bilobectomy	0	2	0	

**Table 5:** Results of the logistic regression analysis (dependent variable: cardiopulmonary complication rate).

Variable	Coefficient	Standard Error	IC	*p* Value
6MWT T1 < 458 m	0.102	0.047	0.01–0.2	0.03
VARIATION T1T0 < 31 m	0.085	0.045	−0.001–0.2	0.05
intercept	0.023	0.034		

6MWT T1: six-min walking test one day before surgery, VARIATION T1T0: difference between the meters walked during the 6MWT for T1 and T0.

## Data Availability

Data supporting reported results can be found within the Electronic Health Record of the Unit of Thoracic Surgery, AOU of Marche, and in the correspondent institutional prospectively maintained database.
